# SIGNOR 4.0: the 2025 update with focus on phosphorylation data

**DOI:** 10.1093/nar/gkaf1237

**Published:** 2025-11-17

**Authors:** Prisca Lo Surdo, Marta Iannuccelli, Klas Karis, Eleonora Meo, Parnian Omidi, Marta Tosoni, Simone Graziosi, Simona Panni, Maria Teresa Fiorillo, Luana Licata, Francesca Sacco, Benjamin M Gyori, Livia Perfetto

**Affiliations:** Department of Biology and Biotechnologies “Charles Darwin”, Laboratory affiliated to Istituto Pasteur Italia-Fondazione Cenci Bolognetti, La Sapienza University of Rome, Rome 00185, Italy; Department of Biology, University of Rome Tor Vergata, Rome 00133, Italy; Khoury College of Computer Sciences, Northeastern University, Boston, MA 02115, United States; Department of Biology, University of Rome Tor Vergata, Rome 00133, Italy; Department of Biology, University of Rome Tor Vergata, Rome 00133, Italy; Department of Biology, University of Rome Tor Vergata, Rome 00133, Italy; Department of Biology, University of Rome Tor Vergata, Rome 00133, Italy; DiBEST Department, University of Calabria, 87036 Rende, Italy; Department of Biology and Biotechnologies “Charles Darwin”, La Sapienza University of Rome, Rome 00185, Italy; Department of Biology, University of Rome Tor Vergata, Rome 00133, Italy; Department of Biology, University of Rome Tor Vergata, Rome 00133, Italy; Khoury College of Computer Sciences, Northeastern University, Boston, MA 02115, United States; Department of Bioengineering, College of Engineering, Northeastern University, Boston, MA 02115, United States; Department of Biology and Biotechnologies “Charles Darwin”, Laboratory affiliated to Istituto Pasteur Italia-Fondazione Cenci Bolognetti, La Sapienza University of Rome, Rome 00185, Italy

## Abstract

The SIGnaling Network Open Resource (SIGNOR 4.0, https://signor.uniroma2.it) is a database of manually curated causal interactions between biological entities. These signaling events are annotated along with their effect—denoting the activation or inactivation of a target entity—and mechanism through which it is mediated, such as phosphorylation, binding, or transcriptional regulation. The data is freely accessible and can be explored as customizable signaling networks, allowing users to adapt them for different modeling purposes. In our latest update (version 4.0), we improved our curation interface to include additional data-validation tools, integrated text-mining-assisted curation, increased our curation content, and expanded the scope of our curation efforts, with a particular emphasis on phosphorylation data. Furthermore, we developed a subdomain of SIGNOR, *PhosphoSIGNOR*, a dedicated user interface designed to enable targeted access to phosphorylation-specific information and network visualization. This expanded dataset allows for a more comprehensive mapping of signaling alterations and their associations with dysregulated cellular processes. The platform enables users to dynamically query phosphosite-specific data, examine context-dependent modifications, and integrate findings with known regulatory mechanisms. The *PhosphoSIGNOR* section of SIGNOR serves as an essential resource for cancer systems biology, offering an intuitive interface for hypothesis generation and mechanistic insights.

## Graphical Abstract

**Graphical Abstract:**
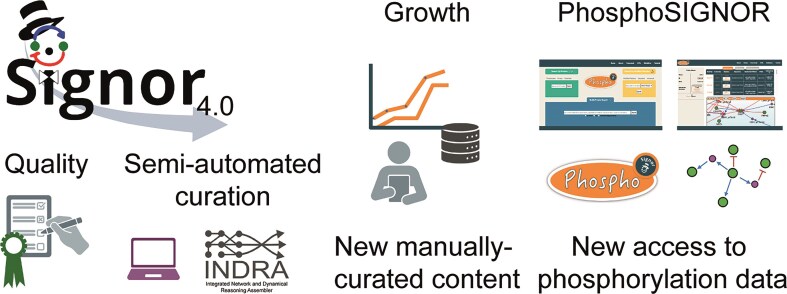

## Introduction

Cells are complex systems whose physiology is regulated by the complex cross-regulation of biomolecules, especially proteins. Indeed, they can respond to environmental stimuli by integrating and propagating received signals to determine targeted responses. The dysregulation of these events might lead to severe diseases such as cancer [1, 2]. In this response a crucial role is played by post-translational modifications (PTMs), especially ubiquitination and phosphorylation, which modulate protein activity and/or stability [3]. Over the last decades, several resources have invested in the digital representation of such signaling events, at different levels of granularity, in the form of pathways or in the form of general interactomes (or both) [4–8]. Among these resources there is SIGNOR, the SIGnaling Network Open Resource (http://www.signor.uniroma2.it) [9]. It is a manually annotated public repository that focuses on the capture, organization, and displaying of signaling interactions represented as binary, causal relationships between biological entities, also known as “activity flow” model [10, 11]. These relationships have the structure “regulatory entity A has a positive/negative effect on a regulated entity B.” Since its development, SIGNOR has had a strong focus on PTM curation, especially (de)phosphorylation reactions mediated by kinases and phosphatases, given their prominent roles in modulating signaling events. Presently, the resource captures signaling relationships between a variety of human biological entities, including biomolecules (proteins, macromolecular complexes, small molecules, etc.), stimuli, and phenotypes. Interactions in SIGNOR are assigned a significance score, ranging from 0.1 to 1, which quantifies the confidence of functional interactions, computed via a principal component regression model [9]. To date, all the interactions form a large and intricate interactome of 13 768 entities connected by 40 940 edges (August 2025).

Here we present the latest update of SIGNOR update (version 4.0). Major improvements include an advanced curation interface and quality check tools as well as the integration of INDRA [12, 13] as a text-mining tool to speed up curation rate. The result of these combined efforts is an increased database content, with a specific focus on PTMs.

Finally, to enable more targeted access to phosphorylation-specific information, we developed a new subdomain of SIGNOR, *PhosphoSIGNOR* (https://signor.uniroma2.it/PhosphoSIGNOR/), a dedicated user interface designed for phosphorylation data browsing and network visualization.

## Results

### Advanced curation tools

A biological data resource relevance is not only based on the amount of stored data but also, and possibly mainly, on the quality standards it follows to ensure accuracy and reliability.

For a resource such as SIGNOR, which is built on the effort of manual curation, it is essential to strive for rigorous quality control measures. The strength of manual curation lies in the more nuanced evaluation of experimental data that is often impossible through an automated process, and the support of an effective curation interface is necessary to assist curators in maintaining precision and efficiency.

To ensure data validation and to speed up the curation process, we made two key developments: first, we implemented routine quality checks (QC) and implemented a novel curation interface with emphasis on protein complexes; second, we designed and implemented a semi-automated curation process making use of the INDRA knowledge assembly system [12, 13].

As part of our QC efforts, we refined the curation interface to catch a wider variety of formatting errors and ensure accurate ID cross-referencing. We also implemented checks for both manual and automated updates in our scoring system and verified key data against UniProtKB for consistency. These implementations now ensure that protein IDs and information (e.g. the protein symbol, function, and sequence) are in sync with the latest UniProtKB release for a more agile interoperability with other resources. These QC also included consistency checks over the controlled vocabulary adopted in SIGNOR and regular checks of the complex entities to avoid the creation of duplicated or highly similar entities and the consistency of the subunit composition. These new steps revealed inconsistencies that had previously been hard to identify—such as complexes that were annotated but whose supporting data evidence had yet to be loaded into our database, or UniProtKB entities that had merged and acquired new IDs—and helped prevent propagation of incomplete or outdated annotations. Consequently, we were able to update our data to reflect the correct information.

**Figure 1.**
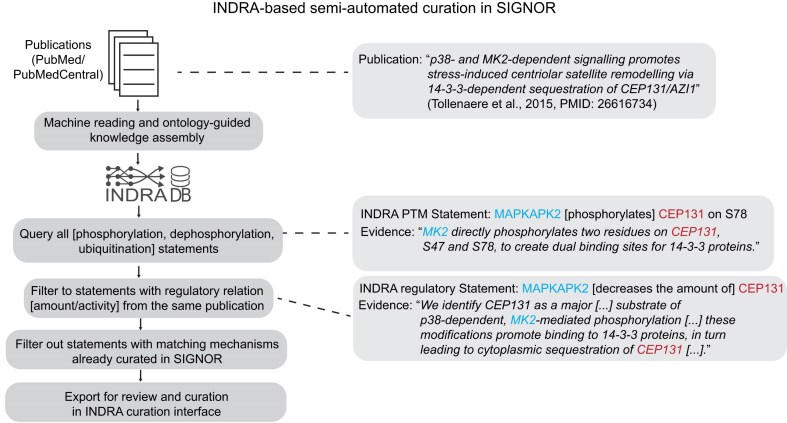
Semi-automated curation of PTM and regulation statements using INDRA. Automated pipeline from source publications processed and assembled using INDRA to exported statements on PTMs and corresponding regulation (left) with a specific example publication and extracted PTM statement and regulation involving MAPKAPK2 and CEP131 (right).

To further accelerate curation, we developed a novel semi-automated workflow building on the INDRA machine reading and knowledge assembly system (Fig. 1). INDRA combines multiple machine reading systems to process literature content and (guided by ontologies) aligns evidence for a given relationship across all supporting sources. We have previously used INDRA to assemble PTM relationships extracted from biomedical abstracts and full-text articles (~25 million abstracts and 10 million full-text articles) into a queriable database. For the purposes of SIGNOR curation, we focused on three PTMs: phosphorylation, dephosphorylation, and ubiquitination, though INDRA can manage a broader class of PTMs that can be targeted in the future. We then implemented several filtering and ranking steps to target curation to relationships that are most often described in literature while being complementary to the existing SIGNOR content. We grouped relationships from INDRA such that both the PTM relationship and its functional regulatory effect can be captured from a single paper, then filtered to relationships that have not yet been curated in SIGNOR (e.g. that MAPKAPK2 dephosphorylates CEP131 on S78, decreasing its stability, a relationship INDRA produced that previously had not been captured in SIGNOR). Finally, we used the INDRA score, which predicts confidence, to prioritize curation, considering, for instance, extraction by multiple independent machines reading systems from different papers. We developed a web-based interface (Supplementary Fig. 1A) through which INDRA statements and the corresponding evidence (specific sentences, linked back to the original article) can be examined and marked as correctly supporting the relationship or marked as correct/incorrect. The code for this web application is available at https://github.com/gyorilab/indra_curation to support INDRA-driven curation efforts more broadly. In the curation interface, statements are sorted and organized by enzyme-target pairs, with modification statements consistently appearing before their associated regulation statements to facilitate efficient curator review of related biological relationships. Curators can mark statements as correct and provide structured comments following a standardized format, such as specifying residue sites, regulatory effects, cellular context, or directness of interaction. These additional text comments are useful if automatically extracted details need to be extended or overridden and are automatically processed for integration into the data export.

In the case of dephosphorylations, we exported a total of 1206 statements from INDRA, covering 370 unique phosphatase-target pairs, often with multiple dephosphorylation statements on distinct phosphosites for a given target, and statements corresponding to the regulatory effect between the phosphatase and its target. In terms of curation, a total of 695 individual evidence sentence-statement pairs were reviewed for correctness.

Subsequently, we developed a pipeline to export curations entered via INDRA’s curation interface into a tabular format directly importable into SIGNOR (Supplementary Fig. 1B). The export process implements a logic that merges curations by consolidating statements with and without site information, combines unique sentences from multiple curations of the same enzyme-substrate-effect relationship, and resolves regulatory effects specified either in modification or regulation statement comments into a unified SIGNOR-compatible representation. The code to generate the statement sets for curation and for exporting curations for ingestion into SIGNOR is available at https://github.com/gyorilab/indra_signor. In the case of dephosphorylations, we exported a total of 235 relationships that were ingested into SIGNOR (an ~20% increase in dephosphorylation relationships). We used the same pipeline and workflow for the semi-automated curation of phosphorylations and ubiquitinations to obtain a total of 313 and 1331 exported relationships, respectively.

### SIGNOR, general content increase

**Figure 2.**
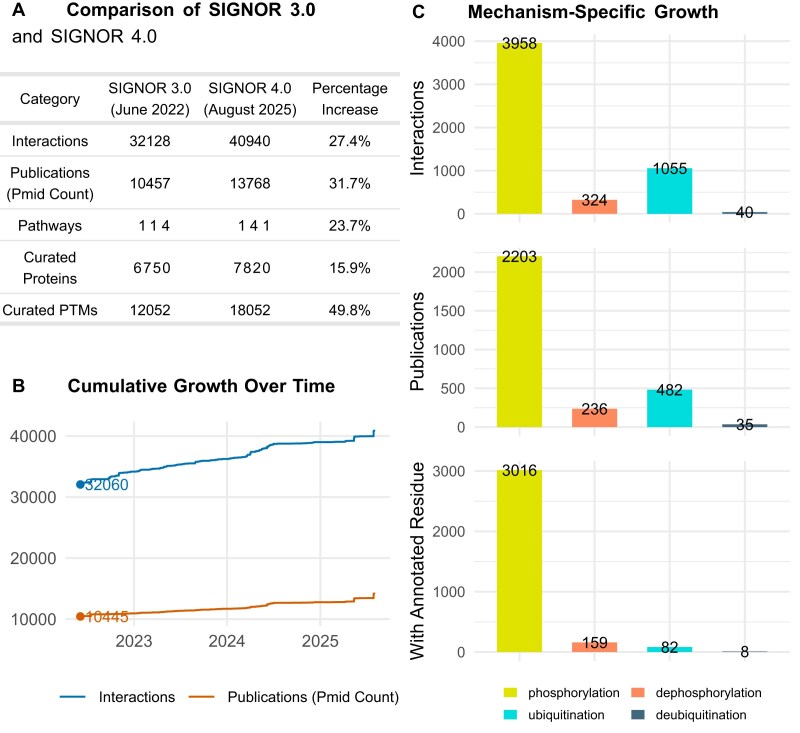
SIGNOR 4.0, content increase. **(A)** Comparison of the causal information captured in SIGNOR 4.0 and 3.0. **(B)** Kinetics of content growth since the publication of SIGNOR 3.0 [9]. The lines represent the growth in the number of annotated interactions and curated manuscripts. **(C)** Histograms showing the number of novel PTM reactions annotated in SIGNOR (top panel), relative number of curated manuscripts (middle panel), and number of PTMs annotated at the residue level (bottom panel).

Following the publication of SIGNOR 3.0 [9] and thanks to the usage of INDRA knowledge assembly system, the SIGNOR curation team has continued the effort aimed at capturing causal information between biological entities. This work resulted in a significant increase in the information content of the resource. More than 8800 new interactions (+27%) were added to the database by curating over 3300 manuscripts (+31%) (Fig. 2A and B), reaching 40 940 curated relationships. Most of the interactions annotated in SIGNOR are between proteins. Additional molecular types or biological entities are, however, also considered: complexes, protein families, miRNA, chemicals, small molecules, phenotypes, stimuli, and others (Fig. 1C). Particularly significant is the increase in proteome coverage. Approximately 38% of the human proteome (7820 proteins) is now integrated in the SIGNOR network (Reference: UniProtKB reviewed human proteome—UP000005640, August 2025) [14].

As discussed in the previous paragraph, over the last release our primary focus has been the annotation of PTMs, namely (de)phosphorylations and (de)ubiquitinations. Presently, SIGNOR stores a total of 15 718 (de)phosphorylations and 2077 (de)ubiquitinations. In Fig. 2C we display the number of interactions curated over the last three years (since previous release in June 2022). As shown, phosphorylation is by far the most frequent annotation in SIGNOR, with >3900 new phosphorylation reactions curated, followed by ~1055 new ubiquitination reactions. Importantly, each interaction in SIGNOR is annotated with the presented molecular mechanism and with the resulting effect (up- or down-regulation) on the target entity. Moreover, whenever possible SIGNOR curators also capture the modified residue (e.g. Ser123) within the target sequence (Fig. 2C, bottom panel). Importantly, 76% and 49% of the phosphorylation and dephosphorylation reactions, respectively, are annotated at the amino acid position level. It is important to underline that we implemented regular updates to ensure that the annotated residues are consistent with the UniProtKB sequence.

### PhosphoSIGNOR, web interface

**Figure 3.**
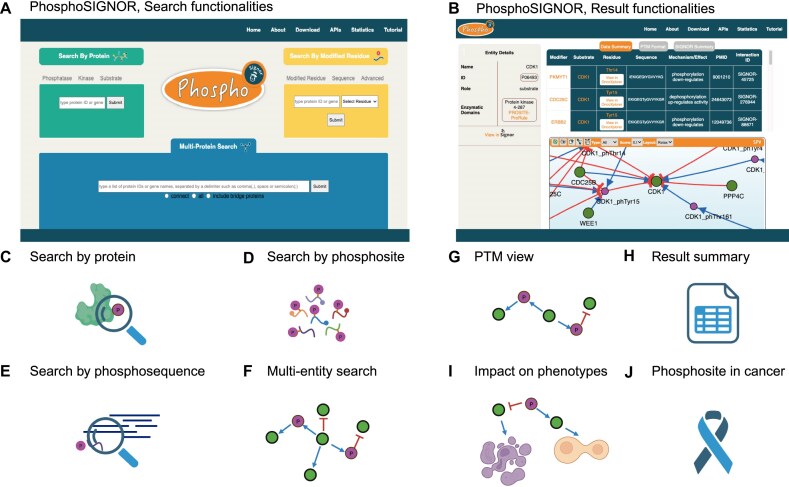
*PhosphoSIGNOR*, web interface. **(A)***PhosphoSIGNOR* home page. **(B)** Example of a result page (query: “CDK1”), summarizing result features. In *PhosphoSIGNOR* it is possible to search by a protein (i.e. a kinase, a phosphatase, or a substrate protein) **(C)**, by a specific phosphosite **(D)**, by a phosphopeptide **(E)**, or to perform a multi-entity query **(F)**. In the result page (de)phosphorylations are displayed as a signed and directed graph, using the “PTM view” (i.e. the phosphosite is an independent node—in pink) **(G)** and summarized as a table **(H)**. For some phosphopeptides, it is possible to retrieve target phenotypes modulated by the peptide **(I)** and their expression level in cancer tissues, as reported in the CPTAC resource [18, 19] **(J)**.

Phosphorylation events have a prominent role within the signaling cascades and therefore are one of the primary focuses when observing signaling dysregulation within the context of disease [3]. Data for the interpretation of such disruptions in the healthy cell signaling pathways can prove to be an essential asset when helpful to identify *ad hoc* treatments or monitoring options. Given the relevance of this subset of SIGNOR’s information, we developed *PhosphoSIGNOR* (https://signor.uniroma2.it/PhosphoSIGNOR/), a dedicated user interface focused entirely on these annotated interactions. Importantly, *PhosphoSIGNOR* features filtered access to SIGNOR’s extensive dataset, including targeted visualization tools (Fig. 3).

*Search Tools—Search by Protein*. The “Search by Protein” tool requires the user to select a type of protein by its role in the regulatory interaction (phosphatase, kinase, or substrate), then enter its gene name or UniProtKB identifier in the input bar (Fig. 3A and C). The built-in autocomplete function filters through entities that are already curated within SIGNOR. Once the query is submitted, the user is redirected to the results page, which will feature all signaling phosphorylation and dephosphorylation interactions found within the SIGNOR dataset that include the chosen entity (Fig. 3B).

Very briefly, in the *PhosphoSIGNOR* section it is possible to retrieve data about >400 kinases and 90 phosphatases (Supplementary Table 1). Each query returns all the (de)phosphorylation reactions that involve the entry enzyme, with a key focus on the substrate protein and more specifically on the modified residue(s).

**Figure 4.**
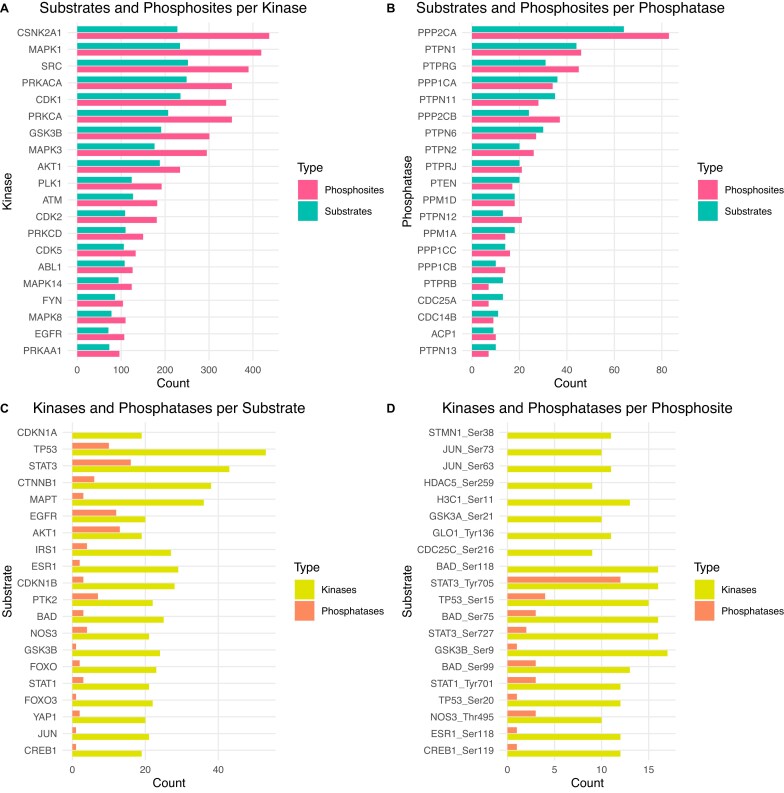
Top 20 entries in *PhosphoSIGNOR*. **(A)** Distribution of target substrates and phosphosites in the top 20 annotated kinases. **(B)** Distribution of target substrates and phosphosites in the top 20 annotated phosphatases. **(C)** Distribution of upstream kinases and phosphatases in the top 20 annotated substrates. **(D)** Distribution of upstream kinases and phosphatases in the top 20 annotated phosphopeptides.

In Fig. 4A we show the top 20 annotated kinases and phosphatases. As shown, for kinases such as CSNK2A1, MAPK1/3, SRC, CDK1, and AKT1, we report >100 substrates and >200 known target residues. This information might be crucial to identify consensus sequences targeted by the kinases. On the other hand, PPP2CA, PTPN1, and PTPRG appear as top annotated phosphatases with >20 substrates and target phosphosites.

Similarly, it is possible to browse 2716 substrate proteins, retrieving all the upstream enzymes that modify specific serine, threonine, and tyrosine residues (Fig. 4B).

As shown, top annotated substrates for de(phosphorylation) include key apoptotic proteins such as TP53 (at Ser15 and Ser20), BAD (at Ser75 and Ser99), and transcription factors such as STAT3 (at Tyr705) and CREB1 (at Ser119).

These findings highlight that phosphorylation data captured in SIGNOR and accessible in the *PhosphoSIGNOR* section allow inspection of the functional significance of PTMs in regulating diverse cellular processes, including, but not limited to, apoptosis and transcriptional control.

*Search tools—Search by Modified Residue*. The “Search by Modified Residue” tool provides various ways to search the dataset through a specific protein residue (Fig. 3D).

Within the “Modified Residue” tab, it is possible to perform a simple search by entering a gene name or UniProtKB ID (using the autocomplete function to select existing entities within the database) and then consulting the “Select Residue” dropdown. This will automatically populate itself with annotated residues of the chosen protein. This will lead the user to a specific results page that will include all the interactions that involve the specific residue.

To support users who do not have a specific residue in mind, the “Sequence” section offers two query options (Fig. 3E). The alignment search allows users to input a sequence and retrieve annotated sequences—along with the corresponding modified residues—from the dataset that match the input with at least 80% identity (Fig. 3E). The quick search option, similar to the “Modified Residue” tab, provides a faster way to browse annotated sequences by selecting a protein and choosing from a pre-populated sequence dropdown menu.

In the *PhosphoSIGNOR* section it is possible to query among 6903 unique phosphosites or phosphopeptides. For each of them, the resource retrieves the upstream kinases and phosphatases that modulate the phosphorylation state, as well as the regulatory role over the host protein (Fig. 4B).

Kinases and phosphatases have opposite effects, as they add and remove phosphate groups, respectively, and might have antagonistic effects on the same target proteins and on downstream cellular pathways. To systematically explore whether our phosphorylation dataset can capture the complexity of this relationship, we set out to compare kinases and phosphatases with the same specificity (Supplementary Fig. 2). This analysis highlights potential functional crosstalk and competition between enzymes that target the same residues. For example, we can confirm a well-known competition between WEE1 and CDC25A [15]. Similarly, we observe that DUSP1/4 and MAP2K1/2 (MEK1/2) have great overlap at tyrosine and threonine sites, suggesting an antagonistic effect on the same pathways. In summary, the observed overlap provides insight into regulatory convergence and may suggest coordinated control or redundancy in signaling networks. (Supplementary Fig. 2)

*Search tools—Multi-Protein Search*. Proteins function within complex signaling networks. To better understand how they regulate one another, it can be helpful to query the database for multiple entities simultaneously. The “Multi-Protein Search” (Fig. 3F) tool supports this functionality, allowing users to input a list of proteins and choose one of three visualization modes:

1. Interactions only between the selected entities;
2. All (phosphorylation/dephosphorylation) interactions involving any of the selected entities; and 
3. Interactions between the selected entities, including bridging proteins that connect them.

*Displaying Results*. Each query in the *PhosphoSIGNOR* section returns results in a tabular format and as a network of causal interactions modelled as “activity-flow” (i.e. represented by a weighted, signed, and directed graph) (Fig. 3G and H). In either case, the *PhosphoSIGNOR* page displays results using the “PTM view” visualization option [16] (Fig. 3G). In the PTM mode, each phosphosite is considered as an independent node, and the enzyme–substrate interaction is split into two sub-interactions: the first connects the upstream kinase (or phosphatase) to the modified residue and is represented as an up- or down-regulation depending on whether the enzyme catalyzes the addition (kinase) or the removal (phosphatase) of the phosphate group. The second edge links the phosphosite to the substrate proteins and reflects the regulatory role of the PTM. As an example, the statement “WEE1 inhibits CDK1 by phosphorylating Tyr15” is split into two interactions: (i) WEE1 up-regulates Tyr15-phosphorylation and (ii) Tyr15-phosphorylation down-regulates CDK1 (Fig. 3B). This type of visualization enables a more granular and mechanistic representation of signaling events, allowing users to derive enzyme activity from the functional consequences of specific PTMs.

*Advanced features of PhosphoSIGNOR*. Phosphorylation events are key to determining a targeted cellular response or phenotype. To identify regulatory associations between individual phosphosites and target biological processes, we used the ProxPath algorithm [17], a computational method designed to infer the functional proximity between any entity in SIGNOR and phenotypic outcomes (Fig. 3I). Here we readapted ProxPath to prioritize phosphosites with a likely impact on cellular processes. Thanks to this, >2500 phosphosites are connected to >150 target phenotypes (Supplementary Table 2). Briefly, users can access this dataset from the advanced tools in the *PhosphoSIGNOR* section homepage. This functionality allows for the identification of key regulatory PTMs that may drive or modulate specific behaviors.

Cancer is a complex disease driven by aberrant signaling pathways, often stemming from dysregulated PTMs of proteins. PTMs serve as key transducers dictating protein function, localization, and interactions [3].

In the big data era, comprehensive proteogenomic profiling of malignant tumor specimens has become common, and large molecular and clinical datasets are made available to the scientific community via public portals, such as the Clinical Proteomic Tumor Analysis Consortium (CPTAC) [18, 19]. To date, the CPTAC provides a comprehensive annotation of PTMs, including phosphorylation, across diverse clinical stages and different cancer types.

To support the identification of PTMs that are dynamically regulated across cancer progression, we decided to link, whenever possible, phosphosites to phosphoproteomic data from 10 tumor types and 1680 patients, available at the CPTAC portal (Fig. 3B and J) [18, 19], and display the results in the form of boxplots. Importantly, this functionality supports the identification of 2278 phosphosites that are dysregulated in cancer samples, which may serve as biomarkers or intervention points in precision oncology (Supplementary Table 3).

*Download and programmatic access*. Phosphoproteomic data is freely available for download (https://signor.uniroma2.it/PhosphoSIGNOR/downloads/) and/or programmatic access (https://signor.uniroma2.it/PhosphoSIGNOR/apis/).

Specifically, in the “Download” page, it is possible to retrieve the complete, manually curated phosphorylation and dephosphorylation datasets in a tabular format. These include all kinase- and phosphatase-substrate relationships.

For more specific queries, it is possible to programmatically access the *PhosphoSIGNOR* section using the “APIs” page. Here, users have the possibility to select specific kinases, phosphatases, or substrate entities. The API can return data in either JSON or TSV format. Depending on the format requested, the output will include biological data regarding annotated phosphorylation and dephosphorylation relations, including entity names and residues, along with mechanisms.

## Discussion

Active since 2015, SIGNOR is a database of signaling information, compliant with the FAIR principles, which ensure data findability, accessibility, interoperability, and reusability [20].

Version 4.0 presents significant additions to the value of the SIGNOR resource. In our latest update, we have implemented a new curation interface complemented with data-validation tools to ensure controlled vocabulary consistency and quality checks. Also, we have carried out an extensive curation effort, with a particular emphasis on phosphorylation data. Indeed, to increase the number of disease networks that can be visualized within SIGNOR, we have integrated the INDRA knowledge assembly system. These efforts have led to a considerable increase in the PTMs coverage. At the present state, our resource captures >15 718 (de)phosphorylation and 2077 (de)ubiquitination events. To improve user experience and (de)phosphorylation data accessibility, we developed a section of SIGNOR termed *PhosphoSIGNOR* (https://signor.uniroma2.it/PhosphoSIGNOR/). *PhosphoSIGNOR* aims at providing access to the high-quality, manually curated phosphorylation data stored in the SIGNOR resource and couples it with novel advanced search functionalities.

Importantly, the *PhosphoSIGNOR* section is conceived to highlight the role of individual phosphorylation sites over the target protein and complex cellular phenotypes. Indeed, phosphosites are at the very core of *PhosphoSIGNOR*, which displays them as individual entries and integrates them in the large SIGNOR interactome, accounting for 40 940 regulatory interactions among proteins, metabolites, complexes, and phenotypes.

Each of the 6903 (de)phosphorylation sites in the *PhosphoSIGNOR* section is connected to the downstream protein targets and to upstream enzymes (information available for 400 kinases and 90 phosphatases), and each interaction is associated with a regulatory effect (up- or down-regulation) and with the literature evidence supporting the interaction. This way of displaying data has the potential to better contextualize phosphoproteomic studies, offering insights into the molecular mechanisms and causality of the regulations. In addition, the *PhosphoSIGNOR* subdomain presents advanced functions: it (i) integrates sequence alignment functionalities that allow users to formulate a query using the phosphosite sequence; (ii) it allows users to display, whenever possible, phosphosite abundance in 10 different tumor types, aggregating information from >1000 patient samples (as available in the CPTAC portal); and (iii) it connects phosphosites to functionally close target cellular phenotypes.

*Current limitations and Next steps*. SIGNOR 4.0 serves as an essential resource for cancer systems biology as well as for signaling networks in neurodevelopmental disorders, such as autism spectrum disorder [21, 22], and autoimmune diseases [23], offering an intuitive interface for hypothesis generation and mechanistic insights. By systematically linking phosphorylation events to cellular phenotypes, it has the potential to accelerate the development of targeted therapies and precision medicine approaches.

Despite this, SIGNOR has limitations. A first limitation is represented by the limited coverage of the resource. To date, only 38% of the human proteome (7820 proteins) is now integrated in the SIGNOR network. Achieving a complete coverage of the human proteome is our mission over the forthcoming years. The continuation of the INDRA-based curation within SIGNOR will be prioritized to speed up the process, considering, for instance, pairs from resources such as PhosphoSitePlus [24]. In addition to (de)phosphorylation reactions, we will also consider within INDRA additional types of PTMs such as (de)acetylation and (de)glycosylation crucial for protein stability, protein subcellular localization, enzyme activity, and transcriptional activity [25, 26].

Another important limitation of our current dataset is its binary interaction structure, which may not fully capture cases where enzymes act as part of multiprotein complexes, such as PP2A/PPP2CA requiring regulatory subunits like PPP2R1A or PPP2R2A for activity and specificity [27, 28]. In SIGNOR, such interactions can be represented by modeling the complex entity as the upstream regulator (e.g. SIGNOR-C132). A systematic inclusion of such complex-based interactions will be prioritized in future updates. This will require extensive manual curation and exploration of human complexes reported in resources such as Complex portal and CORUM [29, 30].

## Supplementary Material

gkaf1237_Supplemental_Files

## Data Availability

SIGNOR 4.0 interaction data are available and freely downloadable at https://signor.uniroma2.it/downloads.php. Furthermore, programmatic access is available through the APIs section at https://signor.uniroma2.it/APIs.php. PhosphoSIGNOR data are available for download at https://signor.uniroma2.it/PhosphoSIGNOR/downloads/ and can also be accessed programmatically via https://signor.uniroma2.it/PhosphoSIGNOR/apis/. All described database features are accessible via the main SIGNOR 4.0 website: https://signor.uniroma2.it/ and the PhosphoSIGNOR subdomain https://signor.uniroma2.it/PhosphoSIGNOR/ SIGNORApp can be freely downloaded from https://apps.cytoscape.org/apps/signorapp.
