# Supplementary material for: SIGNOR 4.0: the 2025 update with focus on phosphorylation data
Source: Nucleic Acids Res. 2025 Nov 17;54(D1):D682–90. doi: 10.1093/nar/gkaf1237 (PMC12807704; doi:10.1093/nar/gkaf1237)
Supplement: gkaf1237_Supplemental_Files [file gkaf1237_supplemental_files.zip › Supplementary_material.pdf]

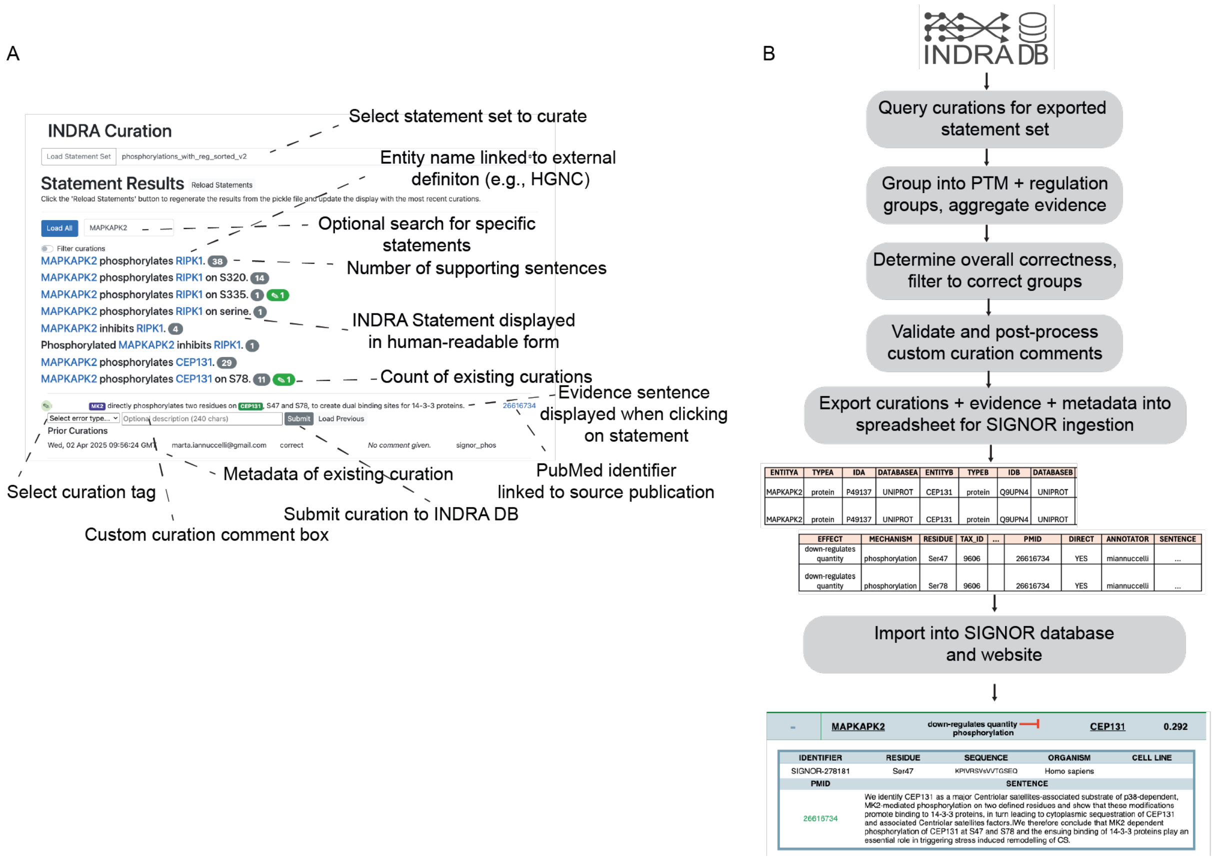

Supplementary Figure 1. INDRA curation in SIGNOR. (A) Annotated screenshot of INDRA curation interface where statements and supporting evidence can be examined and curated for correctness. The expanded statement corresponds to the example from in panel A. (B) Retrieval, post-processing and export of curations added to the INDRA DB for ingestion into the SIGNOR database and website continuing with the example from **Figure 1** and from (**B**).

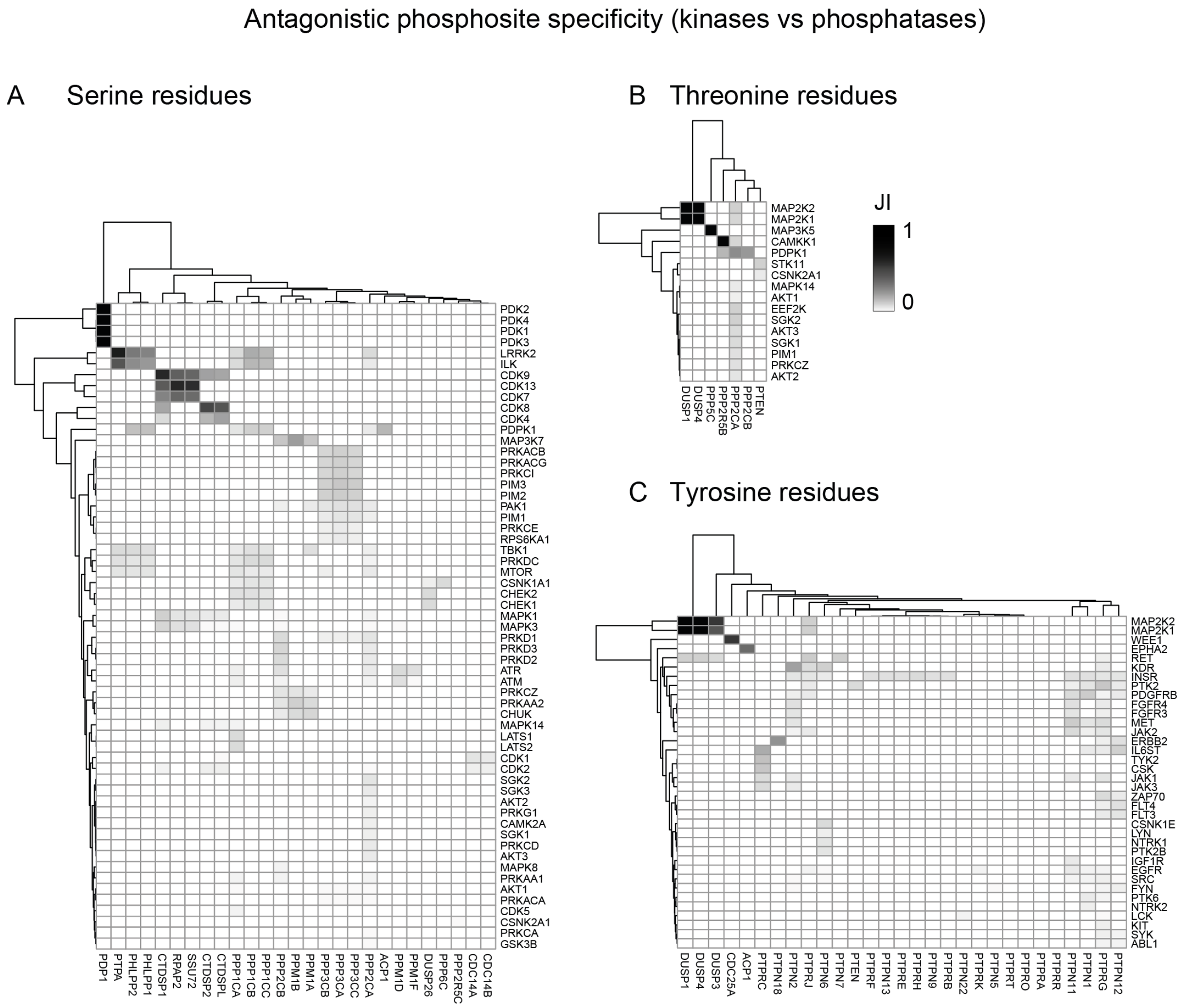

Supplementary Figure 2. Antagonistic specificity between kinases and phosphatases. Heatmap showing the number of shared target Serine **(A)**, Threonine **(B)** and Tyrosine **(C)** residues (displayed as Jaccard's index), between kinases (rows) and phosphatases (columns).
